# Effects of appropriate low-temperature treatment on the yield and quality of pigmented potato (*Solanum tuberosum* L.) tubers

**DOI:** 10.1186/s12870-024-04951-7

**Published:** 2024-04-11

**Authors:** Bi-Cong Chen, Xiao-Jie Wu, Hua-Chun Guo, Ji-Ping Xiao

**Affiliations:** College of Agronomy and Biotechnology, Yunnan Agricultural University, No.95 Jinhei Road, Panlong District, Kunming City, Yunnan 650051 China

**Keywords:** Anthocyanin, Antioxidant substances, Agronomic traits, Low temperatures, Pigmented potatoes, Structural genes

## Abstract

Temperature is one of the important environmental factors affecting plant growth, yield and quality. Moreover, appropriately low temperature is also beneficial for tuber coloration. The red potato variety Jianchuanhong, whose tuber color is susceptible to temperature, and the purple potato variety Huaxinyangyu, whose tuber color is stable, were used as experimental materials and subjected to 20 °C (control check), 15 °C and 10 °C treatments during the whole growth period. The effects of temperature treatment on the phenotype, the expression levels of structural genes related to anthocyanins and the correlations of each indicator were analyzed. The results showed that treatment at 10 °C significantly inhibited the potato plant height, and the chlorophyll content and photosynthetic parameters in the leaves were reduced, and the enzyme activities of SOD and POD were significantly increased, all indicating that the leaves were damaged. Treatment at 10 °C also affected the tuberization of Huaxinyangyu and reduced the tuberization and coloring of Jianchuanhong, while treatment at 15 °C significantly increased the stem diameter, root-to-shoot ratio, yield and content of secondary metabolites, especially anthocyanins. Similarly, the expression of structural genes were enhanced in two pigmented potatoes under low-temperature treatment conditions. In short, proper low temperature can not only increase yield but also enhance secondary metabolites production. Previous studies have not focused on the effects of appropriate low-temperature treatment during the whole growth period of potato on the changes in metabolites during tuber growth and development, these results can provide a theoretical basis and technical guidance for the selection of pigmented potatoes with better nutritional quality planting environment and the formulation of cultivation measures.

## Introduction

Potato is one of the important food crops crop worldwide. Because of its good adaptability to various environments, it has the excellent characteristics of cold resistance, drought resistance and barren resistance. Potato is also a good cash crop and plays an important role in food security and nutrition. Pigmented potato is a variety of common cultivated potato. The skin or flesh of pigmented potato tubers can be red, orange, purple, blue or black [1], and the advantage of pigmented potato tubers compared with regular potato tubers is not only their brightly pigmented skin but also the higher nutritional value of their flesh [2]. Because of their relatively high anthocyanin content, pigmented potato tubers contain more polyphenolic compounds and more antioxidant substances than ordinary potato tubers [3, 4] and can be used as plant source materials to obtain natural anthocyanins [5]. Compared to regular potato tubers, pigmented potatoes offer enhanced nutritional and medicinal benefits, boasting higher levels of biological activity. They are considered a healthier choice for consumers and hold a special position in the potato processing industry due to their unique characteristics and potential health-promoting properties [6].

Temperature is one of the important environmental factors affecting plant growth and development as well as yield and quality [7]. Long-term low temperatures will hinder plant development and even decrease yield [8, 9]. Potato originated in the Andes Mountains of South America, where they adapt to the cold climate. For most commercial potato varieties, the suitable temperature for growth is 14–22 ℃ [10]. However, too low-temperature will also hinder the growth of potato. Potato seedlings stop growing when the temperature is below 7 °C [11], and the growth temperature of 4 °C leads to restriction of the carbohydrate oxidation pathway in tubers [12]. Meanwhile, other research results have shown that for cool-season crop species such as potato, proper low temperature can increase the proportion of carbon allocated to potato tubers [13] and the allocation of assimilates [14], improve carbohydrate metabolism [12] and thus improve the yield of potato. The optimal temperature for inducing the development of potato tubers is 15 °C [15] A decrease in temperature will increase the proportion of tubers during the expansion process, but when the temperature is reduced to 4 °C, the respiration of tubers rapidly decreases to the minimum and the development speed slows down, when the temperature is below − 2 °C, the tuber development stops [16, 17]. Nolan’s data showed that potato tuber yield was the highest at moderately low temperatures [18]; for some special potato varieties, appropriate temperature reductions can even increase yield several times [19]. Many plant species show adaptation to low-temperature environments through the accumulation of more polyphenolic compounds. Moreover, red- and purple-fleshed potato varieties contain higher levels of polyphenolic compounds than light-pigmented (yellow) potato varieties [2, 3, 20]. At the same time, total phenolic and flavonoid contents were found to be significantly increased in purple-fleshed and red-fleshed potato tubers of the same variety grown in cooler regions [21, 22]. Studies have shown that low-temperature environments cause various plants to exhibit significant accumulation of anthocyanins [23–26]; at the same time, production has shown that the anthocyanin content increased significantly in tubers of the same variety of purple- and red-fleshed potato plants grown in areas with relatively low temperatures [21, 22].

Low-temperature stress causes a series of changes in the plant membrane system, physiological metabolism and protective enzyme system. Studies have shown that some plant species develop cold resistance under prolonged low-temperature stress, such as through changes in osmotic adjustment substances and enzyme activities to resist low-temperature stress [27]. At the same time, low temperature reduces the chlorophyll content of plants [28], inhibits the growth and development of plants, and may cause a series of symptoms, such as leaf curling, stem damage, and slow growth. Low temperature can also affect the photosynthesis of plants [29], such that excessive accumulation of reactive oxygen species (ROS) damages the cell membrane system, resulting in the production of malondialdehyde (MDA) [30, 31]. With decreasing treatment temperature, the longer the low-temperature treatment time was, the more MDA accumulated in the plants. Therefore MDA is an important indicator of membrane system damage [32], and the adaptability of plants to low temperatures can be reflected by the MDA content [33]. Superoxide dismutase (SOD) and peroxidase (POD) can efficiently remove harmful substances such as active oxygen compounds and free radicals in plants to reduce damage [34, 35]. Many studies have shown that the activities of peroxidase and superoxide dismutase in most plants are closely related to the cold resistance of these plants under low temperature conditions [35, 36].

Appropriate low temperature can affect plants to accumulate secondary metabolites [37], which contains some nutrients, during the process of adaptation to the environment. Studies have confirmed that confirm that at the early stage of low-temperature treatment, the ascorbic acid (ASA) metabolic system in potato leaves becomes stimulated; the ASA content first increases, peaks and then decreases [38]; and the content of chlorogenic acid also increases by approximately 15% after potato tubers are stored at low-temperature [39]. Low-temperature stimulation is also an important condition for promoting anthocyanin synthesis in a variety of plants [40], and results have shown that low temperatures can affect flesh color [41]. The optimal temperature points for tissue coloration in different species are not the same as those for cultivars, and the temperature difference between day and night has a large effect on fruit and root tuber coloration. Moreover, the larger temperature difference is conducive to the accumulation of sugar, thus promoting the synthesis of most plant anthocyanins [42]. Low temperature has been reported to promote anthocyanin biosynthesis in, for example, red skinned grape pericarp [24] and in the leaves of Vicia farensia [23], thereby improving plant adaptation to low-temperature environments.

The synthesis of anthocyanins is regulated by structural genes and transcription factors. The potato anthocyanin biosynthetic pathway can be divided into three periods [43, 44]. First, phenylalanine forms phenylacrylic acid under the action of phenylalanine ammonia lyase (*StPAL*), undergoes flavonoid metabolism, converts to naringenin under the combined action of chalcone synthase (*StCHS*) and chalcone isomerase (*StCHI*), and finally forms various types of pigments under the catalysis of dihydroflavonol 4-reductase (*StDFR*). flavonoid 3,5-hydroxylase (*StF3’H*) and flavonoid 3,5-hydroxylase (*StF3’5’H*) are the main enzymes that contribute to the diversification of anthocyanins, and anthocyanin synthetase (*StANS*) and UDP glucose flavonoid 3-o-glucosyltransferase (*StUFGT*) are the last two enzymes in the anthocyanin synthesis pathway of potato to ultimately form stable anthocyanins. Low temperature was confirmed to enhance anthocyanin biosynthesis in blood oranges through enhanced upregulation of major structural genes and transcription factor-coding genes that regulate the activity of the flavonoid pathway [25]. After low-temperature treatment, almost all the genes directly involved late anthocyanin biosynthetic genes (*LBGs*) were more highly expressed in purple head Chinese cabbage (*Brassica rapa* L.) [26]. This treatment resulted in a darker epidermis, and the expression levels of *CHS3*(Chalcone Synthase 3), *F3’H1*(Flavonoids 3 ‘- hydroxylase 1), *MYBA1(Myeloblastosis oncoprotein A1)* and *UFGT* (Glucose-flavonoid 3-o-glucosyltransferase)increased significantly [45]. This indicated that low temperature may regulate the content of anthocyanins through regulation of the expression of structural genes.

According to practical production experience, the color of the same pigmented potato variety planted at high altitudes is significantly darker than that at medium and low altitudes in the same season. It is presumed that low temperature has an important stimulating role in the synthesis of anthocyanins in pigmented potato. Previous studies on the response of potato to low-temperature treatment have focused mainly on the changes in physiological and biochemical indicators of potato leaves under extreme low-temperature treatment and their correlations or on the changes in metabolite composition during the storage of potato tubers [7, 39, 46].

This study focused on the effects of different temperature treatments on the yield and quality of tubers of two pigmented potato varieties, it also explored the effect of temperature on plant growth and material accumulation. These results will give important guiding significance for improving the economic value of pigmented potato and the selection of suitable cultivation environments.

## Materials and methods

### Potato materials

The red potato was named Jianchuanhong, and the purple potato was named Huaxinyangyu. At 25 days after propagating tissue culture-produced seedlings, the ramets were transplanted into pots, after which the pots were placed under three different temperature treatments. Generally, the optimum soil temperature for normal growth of potato tubers is 15–18 °C, and temperatures below 10 °C severely inhibit potato tuber growth [47], so the light/darkness treatment temperature gradients were set as CK (20 °C), 15 °C, and 10 °C, in growth chambers with a light intensity of 11,000 lx and a photoperiod of 12/12 h (light/darkness). The total growth period of all the plants was 96 days.

At 24, 48 and 72 days after treatment, the last four leaves were collected from plants treated at different temperatures. Each treatment combined three tubers with three biological replicates, and the tubers treated with CK were used as control.

Potato tubers began to appear at 48 days of treatment and were harvested every 24 days thereafter until the end of the growth period. Three replicates were mixed into one sample, and portions of the tubers were measured for secondary metabolites.

The photosynthetic rate was measured by a portable photosynthetic system (LI-6400, LI-COR, NE, USA) after 48 days of treatment because the aboveground plant growth and tuber growth were parallel.

### Plant morphological indexes and tuber production parameters

To determine the growth of potato plants under different temperature treatments, the height, stem thickness, and leaf area of individual plants from the 3 temperature treatments were quantified. The number, weight and root-to-crown ratio of the tubers produced per plant were assessed.

### Determination of chlorophyll content

The chlorophyll content was determined by spectrophotometry [48]. A total of 0.1 g of each sample was weighed and placed into a 25 mL test tube. Then, 20mL of 95% ethanol was added, the test tube was thoroughly shaken, and the tube was incubated overnight in the dark at room temperature until the color turned white. The extract was fixed in 95% ethanol to scale and transferred to a 1 cm cuvette, after which the absorbance was measured at wavelengths of 665, 649 and 470 nm. The amount of chlorophyll a (mg/L) was 13.95*A665-6.88*A649; the amount of chlorophyll b (mg/L) was 24.96*A649-7.32*A665; the total chlorophyll concentration was calculated as chlorophyll a (mg/L) + chlorophyll b (mg/L); and the chlorophyll pigment content (mg/g) was calculated as (total chlorophyll concentration (mg/L) * total extract (ml))/(leaf sample weight (g) * 1000).

### Determination of relative conductivity

The conductance method was used to determine the relative conductivity [49]. A total of 0.2 g of fresh leaves was weighed, cut into uniform sizes, and put into a 10 mL test tube. Afterwards, 10 mL of deionized water was added, and the material was incubated for 4∼5 h at room temperature. The conductivity was subsequently measured with a DDs-11 conductivity meter. The material was then placed in a boiling water bath for 15 min and allowed to cool to room temperature. Afterwards, the total conductivity was measured to express the degree of cytoplasmic membrane permeability based on the relative conductivity. The relative electrolyte exudation rate (%) was equal to L1 (immersion solution conductivity)/L2 (conductivity after boiling) × 100%.

### Determination of MDA content

The thiobarbituric acid (TBA) color development method was used [50]. A 0.2 g sample was obtained, 10% trichloroacetic acid (10 mL) was added, and the sample was ground until homogenized. Then, 5 mL of supernatant and 5 mL of 0.6% TBA were mixed together, boiled in a water bath at 100 °C for 30 min, cooled quickly on ice and centrifuged again to remove the supernatant, after which the absorbance values were measured at 450, 532 and 600 nm.

### Determination of antioxidant enzyme activity

SOD enzyme activity was measured by the photoreduction method [51] via nitroblue tetrazolium. A total of 0.2 g of sample tissue was ground into a homogenate in phosphate buffer (pH 7.8), and the supernatant was centrifuged. EDTA, methionine and riboflavin were added, after which the contents were shaken and mixed. The sample was then placed in a growth chamber with a light intensity of 4000 lx at 25 °C for 25 min. The absorbance was then measured at 560 nm.

POD enzyme activity was measured by the guaiacol method [52]. A total of 0.2 g of sample tissue was ground into a homogenate in phosphate buffer (pH 7.0), after which the supernatant was centrifuged and added to the reaction mixture (phosphate buffer, 2% hydrogen peroxide, 1% guaiacol mixture) to determine the absorbance at 470 nm. The readings were taken at 10 s intervals for 2 min.

### Determination of total phenol, flavonoid and anthocyanin contents

The total phenol content was determined under alkaline conditions by the use of a G0117F kit (Grace Biotechnology Co., Ltd., Suzhou, China). The reduction of tungsten-molybdic acid by phenolics produces a blue compound with a characteristic absorption peak at 760 nm, and the absorbance value was read at 760 nm to calculate the total phenol content.

The flavonoid content was determined by [53] weighing 0.5 g of lyophilized potato tissue, adding 1% hydrochloric acid and methanol, and grinding until the mixture was homogenized. Afterwards, the contents were transferred to a 25 mL volumetric flask, and the volume was brought to scale. After ultrasonication, the supernatant was centrifuged and sequentially added to a 5% sodium nitrite solution, a 10% aluminum nitrate solution and a 4% sodium hydroxide solution and shaken well. Finally, the sample was diluted to scale with distilled water and read spectrophotometrically at 510 nm to determine the absorbance.

The anthocyanin content was determined by weighing 0.5 g of lyophilized potato sample, adding 10 mL of anthocyanin extract (95% ethanol mixed with 1.5 mol/L hydrochloric acid), and grinding until homogenization. Afterwards, the mixture was subjected to ultrasonic treatment and centrifugation to extract the supernatant; 5 mL of anthocyanin extract was then added to the filter residue, which was subjected to ultrasonic treatment followed by centrifugation to extract the supernatant [54]. The absorbance values of the extracts were measured at visible-spectrum absorption wavelengths of λvis-max (Jianchuanhong, 512 nm; Huaxinyangyu, 525 nm) and 700 nm.

### Determination of chlorogenic acid content

A total of 0.1 g of lyophilized sample was weighed. Afterwards, 10 mL of 70% ethanol was added to dissolve the sample, which was then heated in a water bath at 85 °C for 1 h. Afterwards, the sample was allowed to cool to room temperature. Five millilitres of the supernatant plus 5 mL of 70% ethanol were subsequently centrifuged, after which the contents were mixed well. The absorbance was measured at 327 nm, and 70% ethanol was used as a blank [55].

### Determination of vitamin C content

The vitamin C content was measured using a G0201f kit (Grace Biotechnology Co., Ltd., Suzhou, China). The measurements are based on the principle that reduced ASA reduces trivalent iron ions to divalent iron ions and reacts with red phenanthroline to form a red complex with a characteristic absorption peak at 534 nm. The color depth is proportional to the content of reduced ASA.

### Fluorescence quantitative PCR

According to the existing studies, the expression of the *StPAL*, *StF3’H*, *StCHS*, *StDFR*, *StANS* and *StUFGT* genes changes significantly after low-temperature treatment [26, 56, 57], so these six structural genes were selected for determination in this study.

qRT‒PCR primers were designed according to the sequences of the potato *StPAL*, *StF3’H*, *StCHS*, *StDFR*, *StANS* and *StUFGT* genes, and the *StGAPDH* gene of potato was used as a housekeeping gene (Table 1). SYBR Premix Ex Taq™ (Bao Bioengineering Co., Ltd., Dalian, China) was used. For qRT‒PCR, a 20 µL reaction mixture was prepared on ice according to the manufacturer’s instructions: the mixture included 1× SYBR Premix Ex Taq™, upstream and downstream primers (0.5 µmol/L each), and approximately 30 ng of cDNA. Each reaction was repeated 3 times, and ddH_2_O was used as a negative control. The PCR program was as follows: predenaturation at 95 °C for 30 s followed by 45 cycles of denaturation at 95 °C for 5 s, annealing at 60 °C for 30 s, and extension at 72 °C for 30 s. After the reactions, the relative expression level was calculated by the 2^−ΔΔCT^ method according to the cycle threshold (Ct) value of the obtained gene.

**Table 1:** Primer sequences used for RT-PCR

Gene	Forward primer (5’–3’)	Reverse primer (5’–3’)	bp	Tm (°C)
*StPAL*	TCGAGGACGAATTGAAGGCT	CAGGGTTGCCACTTTCAAGC	75	60
*StCHS*	TGATTGAAGCATTCCAACCAATAG	GTTCAACTTGGTCAAGAATTGCC	96	60
*StF3’H*	CGTCAACGTTTGGGCCATTT	TTCACCTCCGGGCAAGAATC	88	60
*StDFR*	ACCCAAAGCACTGCAGAC	CTGGTGCATTCTCCTTGCC	88	60
*StANS*	TGAGTCCCTCCTAAAGGCCA	TCATGTTTCCGCGTTCTCCA	86	60
*StUFGT*	TGAATGCGTTTGCGTTGGTT	ACATGCTCATATTTCCCCACGA	82	60
*StGAPDH*	ATGAAGGACTGGAGAGGTGG	GAAAATGCTTGACCTGCTGT	75	60

### Statistical analysis

The means, standard deviations and standard errors were calculated in Excel. The graphs of the correlation analysis experiment were constructed using Origin [58]. All the data are the means of three biological replicates. Analysis of variance (ANOVA) was performed in SPSS. Differences were considered statistically significant when *P* < 0.05.

## Results and discussion

### Effects on plant growth and leaf development

**Fig. 1:**
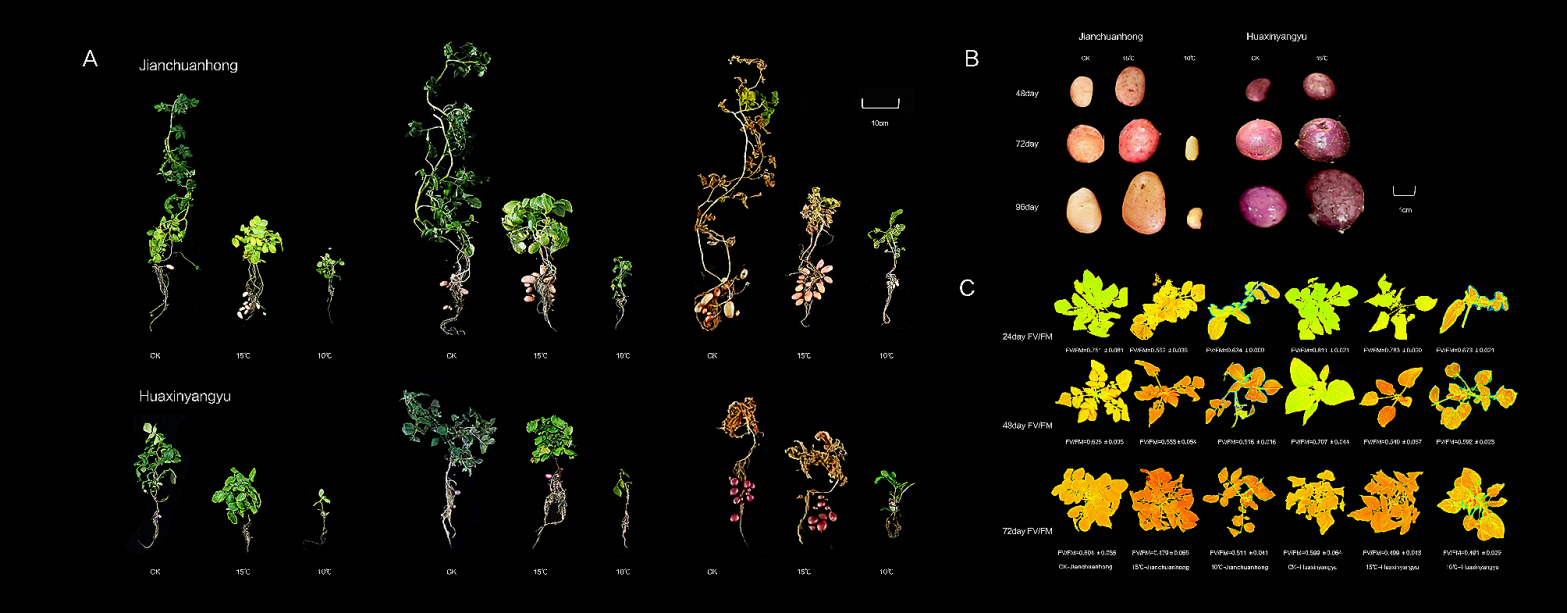
Plant and tuber growth of two potato varieties under different temperature treatments. Changes in plants of two potato varieties after different temperature treatments. B. Changes in tubers of two potato varieties after different temperature treatments. **C**. Leaf injury degree of two varieties of potato after different temperature treatments. The darker the leaf color, the smaller the value of FV/FM, and the greater the degree of leaf damage

In terms of morphology, plants subjected to long-term appropriate low-temperature were compared with control plants (Table 2; Fig. 1). Both potato varieties showed significant changes after low-temperature treatment compared with the control treatment, and the height of the treated plants was significantly lower than that of the control plants, but the stem diameter and leaf area were greater than those of the control plants. Both Jianchuanhong and Huaxinyangyu produced obvious pigmented potato tubers beginning at 48 days after treatment (Fig. 1B), and the degree of leaf damage significantly increased in plants subjected to low temperature during tuber formation compared with that in the control plants (Fig. 1C). The characteristics and sources of two pigmented potato varieties were showed in Table 3.

**Table 2:** Analysis of various growth parameters of potato plant leaves and tubers in response to low temperature. -- means no data. The values represent the means ± SDs; *n* = 3. The means with different letters are significantly different from each other (*P* < 0.05)

Processing period	Variety	Temperature (°C)	Plant height (cm)	Stem diameter (mm)	Leaf area (cm^2^)	Tuber number per plant	Tuber fresh weight per plant (g)	Root-to-shoot ratio
24 day	Jianchuanhong	CK	34.25 ± 3.14 c	2.19 ± 0.26 d	0.88 ± 0.37 f	--	--	--
		15 °C	18.23 ± 2.05 e	4.18 ± 0.35 c	11.47 ± 6.47 de	--	--	--
		10 °C	7.02 ± 0.41 f	1.40 ± 0.38 d	13.76 ± 3.45 cde	--	--	--
	Huaxinyangyu	CK -	16.29 ± 1.37 e	2.71 ± 0.54 bc	3.41 ± 1.98 g	--	--	--
		15 °C	11.70 ± 2.05 f	2.88 ± 0.50 bc	20.95 ± 3.95 cd	--	--	--
		10 °C	4.84 ± 0.92 i	1.45 ± 0.33 d	14.87 ± 6.78 ef	--	--	--
48 day	Jianchuanhong	CK	70.83 ± 1.56 b	4.01 ± 0.81 c	3.40 ± 0.86 f	2.33 ± 0.58 d	4.00 ± 0.43 d	1.36 ± 0.21 a
		15 °C	26.25 ± 1.66 d	4.44 ± 0.56 c	20.46 ± 0.79 bc	4.67 ± 1.15 d	7.63 ± 0.87 c	6.18 ± 1.59 b
		10 °C	8.65 ± 0.37 f	1.73 ± 0.36 d	14.99 ± 3.12 cd	--	--	--
	Huaxinyangyu	CK	26.27 ± 2.52 b	3.61 ± 0.61 bc	8.72 ± 2.27 fg	3.67 ± 1.15 c	3.03 ± 0.94 c	0.56 ± 0.13 c
		15 °C	20.17 ± 1.04 d	4.07 ± 0.80 b	39.74 ± 4.18 b	7.00 ± 1.73 bc	6.58 ± 1.08 c	1.68 ± 0.08 a
		10 °C	6.99 ± 0.42 h	1.51 ± 0.14 d	20.96 ± 4.46 cd	--	--	--
72 day	Jianchuanhong	CK	87.57 ± 6.46 a	5.48 ± 0.70 b	7.18 ± 0.96 ef	5.33 ± 0.58 d	7.28 ± 1.48 c	1.48 ± 0.05 a
		15 °C	29.43 ± 4.55 d	6.12 ± 0.79 a	34.76 ± 5.87 a	11.67 ± 2.52 c	15.72 ± 1.70 a	2.91 ± 0.55 a
		10 °C	9.24 ± 0.62 f	2.01 ± 0.27 d	25.82 ± 8.94 b	--	--	--
	Huaxinyangyu	CK	33.05 ± 1.75 a	3.91 ± 0.22 b	16.84 ± 3.66 d	4.67 ± 1.53 c	5.64 ± 1.52 c	1.20 ± 0.02 b
		15 °C	22.73 ± 2.64 c	5.37 ± 1.30 a	56.34 ± 9.38 a	9.67 ± 2.08 b	13.40 ± 2.08 b	1.82 ± 0.14 a
		10 °C	9.52 ± 1.31 g	2.04 ± 0.49 cd	26.24 ± 8.66 c	--	--	--
96 day	Jianchuanhong	CK	--	--	--	15.33 ± 1.53 b	11.77 ± 2.00 b	1.52 ± 0.10 a
		15 °C	--	--	--	19.67 ± 1.53 a	17.65 ± 0.47 a	15.12 ± 1.10 a
		10 °C	--	--	--	--	--	--
	Huaxinyangyu	CK	--	--	--	6.33 ± 1.15 bc	6.46 ± 1.21 c	0.33 ± 0.03 c
		15 °C	--	--	--	13.33 ± 1.53 a	20.39 ± 6.66 a	2.09 ± 016 a
		10 °C	--	--	--	--	--	--

**Table 3:** Tuber traits and sources of the two pigmented potato cultivars

Variety	Skin color	Potato flesh color	Potato shape
Huaxinyangyu	Purple	Light yellow with purple ring	Oblate oval
Jianchuanhong	Light Red	Light yellow with pink ring	Reniform
Taste	Potato Skin	Source and Cultivation type	
Soft and sweet	Rough	Yunnan Local Characteristic Varieties Tetraploid cultivar	
Delicate and smooth	Smooth	Yunnan Local Characteristic Varieties Tetraploid cultivar	

**Fig. 2:**
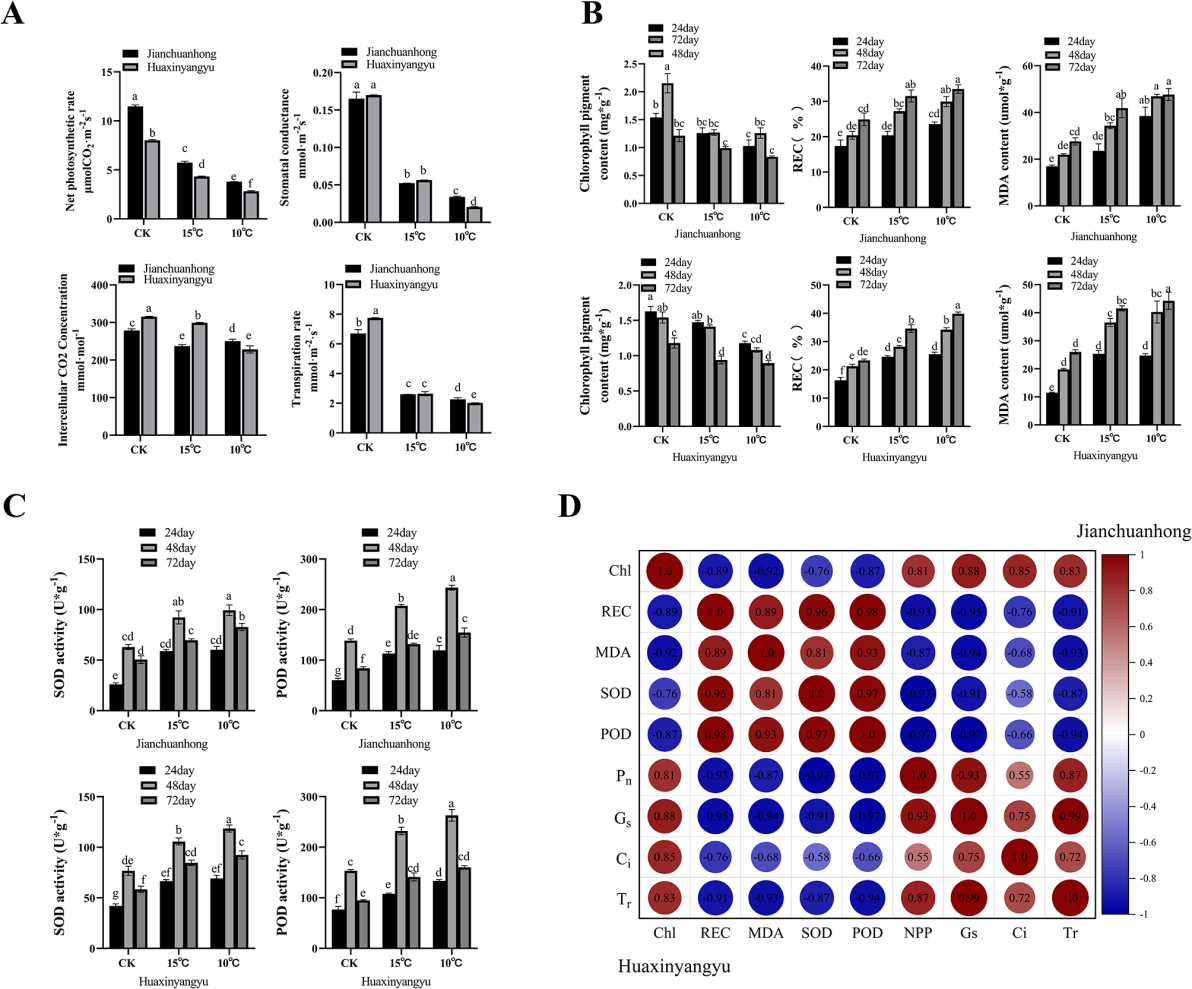
Physiological indicators and correlation analysis of potato leaves. **(a)** Measurement of photosynthetic data of potato leaves after proper low-temperature treatment for 48 days. **(b)** Changes in the chlorophyll content, relative conductivity and MDA content in leaves of two potato varieties under low-temperature treatment. **(c)** Changes in SOD activity and POD activity in the leaves of two potato varieties under low-temperature treatment. **(d)** Correlation analysis of physiological indexes of potato leaves. The red color represents a positive correlation, and the blue color represents a negative correlation. REC means relative conductivity; Chl means chlorophyll; F_n _means net photosynthetic rate, G_s_ means stomatal conductance; C_i_ means intercellular CO2 concentration; T_r_ means Transpiration rate. The data are the mean ± SD; *n* = 3; the larger the value, the more significant it is (*P* < 0.05)

Explore whether the aboveground plant growth of potato was inhibited, we measured the net photosynthetic rate, stomatal conductance, intercellular carbon dioxide concentration and transpiration rate of leaves at 48 days (Fig. 2A). Compared with those in the control leaves, all the values were significantly reduced, and the reduction was more significant at 10 °C. Moreover, compared with that of the control plants, the chlorophyll content in the leaves of both potato varieties showed a tendency to decrease with prolonged low-temperature treatment (Fig. 2B), and the lower the temperature was, the higher the degree of leaf chlorophyll loss. Compared with those of Huaxinyangyu, the performance of Jianchuanhong decreased more significantly. The relative conductivity increased, and the MDA content increased significantly in the leaves; moreover, the performance of Huaxinyangyu increased significantly (Fig. 2B).

Generally, the optimal soil temperature range for the normal growth of potato tubers is 15–18 °C, with temperatures below 10 °C and above 30 °C severely inhibiting tuber growth [59]. In our experiment, exposing the aboveground parts of the two potato varieties to 10 °C resulted in slow growth, while the belowground parts struggled to form tubers. Specifically, Jianchuanhong started producing colorless, small potatoes after 72 days of treatment, with colorless potato skins produced by the end of the growth period. Moreover, Huaxinyangyu did not develop tubers throughout the growth period. These findings suggest that 10 °C may not support the growth and development of these potato varieties optimally.

Chlorophyll, a key component for photosynthesis in plants, reflects productivity and adaptability to some extent [60]. Under low-temperature treatment, it also indicates cold resistance; potato leaves undergo membrane lipid peroxidation, and the malondialdehyde (MDA) content reflects plant adaptation to low temperature stress [28]. Lower temperatures can inhibit enzyme activity and hinder enzymatic reactions, leading to increased chlorophyll degradation and weakened synthesis, thereby reducing the total chlorophyll content and affecting the photosynthetic rate [61]. Despite decreased net photosynthetic rates in both varieties, our data showed that exposing them to 15 °C significantly increased yields (Table 1), likely due to enhanced nutrient accumulation in potato tubers. Notably, the root-to-shoot ratio increased significantly after low-temperature treatment, as did the tuber number and weight. This may be attributed to low temperature inhibiting leaf development to affect photosynthesis, and redirecting resources to storage organs (tubers) for reproduction and stress resistance [26, 62]. Therefore, our study concluded that treatment at 15 °C can substantially improve yield.

### Effects on antioxidant enzyme capacity in potato leaves

In the measured leaves of the three periods, the superoxide dismutase (SOD) activity and peroxidase (POD) activity of the leaves of the two pigmented potato varieties treated at 15 °C were higher than those of the control group throughout the whole process (Fig. 2C). Among them, the difference in enzyme activity in Jianchuanhong leaves at 72 days was the most significant (SOD activity increased by 35%, and POD increased by activity 57%). After low-temperature treatment, the dynamic balance of the active oxygen metabolism system in plants becomes changed, and the activities of various enzymes in the plant are significantly altered. These fluctuations in enzyme activities are related to the plant’s response to temperature [63].

Xu’s research demonstrated that physiological indicators such as SOD and POD activity increase following low-temperature treatment in different potato varieties [64], with POD activity showing a significant correlation with temperature. Analysis of enzyme activities in potato leaves from Jianchuanhong and Huaxinyangyu under low-temperature treatment revealed a consistent trend in SOD and POD activities between the two varieties. This indicates that both 15 °C and 10 °C exert a certain impact on the growth and development of leaves in these potato varieties.

### Correlation analysis of physiological indexes of potato leaves

We conducted a correlation analysis on the leaf physiological indicators of two pigmented potato varieties (Fig. 2D) and found that the chlorophyll content was extremely significantly negatively correlated with the relative conductivity, MDA content, SOD activity and POD activity, and was extremely significantly positively correlated with photosynthetic indicators. Combined with the increase with time and decrease in temperature shown in Fig. 2-B, the chlorophyll content decreased with time. It shows that leaves are indeed slightly affected by lower temperatures. Moreover, 15 °C and 10 °C inhibit the growth and development of leaves and reduce the photosynthetic capacity of two pigmented potato leaves. As shown in Fig. 1 and Table 2, the plants treated at 10 ℃ exhibited weak growth and difficulty producing potatoes. The root-to-shoot ratio of the plants increased, the number and weight of the tubers increased, and the color of potato skin significantly deepened under the 15 °C low-temperature treatment. We believe that 15 °C low-temperature treatment can increase the yield of two types of pigmented potatoes.

Thus, the obtained data showed that the growth, tuber yield and antioxidant enzymes of both varieties of plants were significantly affected by prolonged and appropriate low-temperature treatment, and based on these results, we further investigated whether low-temperature treatment would have a positive effect on the contents of some nutrients and secondary metabolites in potato plants.

### **Effects on antioxidant substances in potato tubers**

**Fig. 3:**
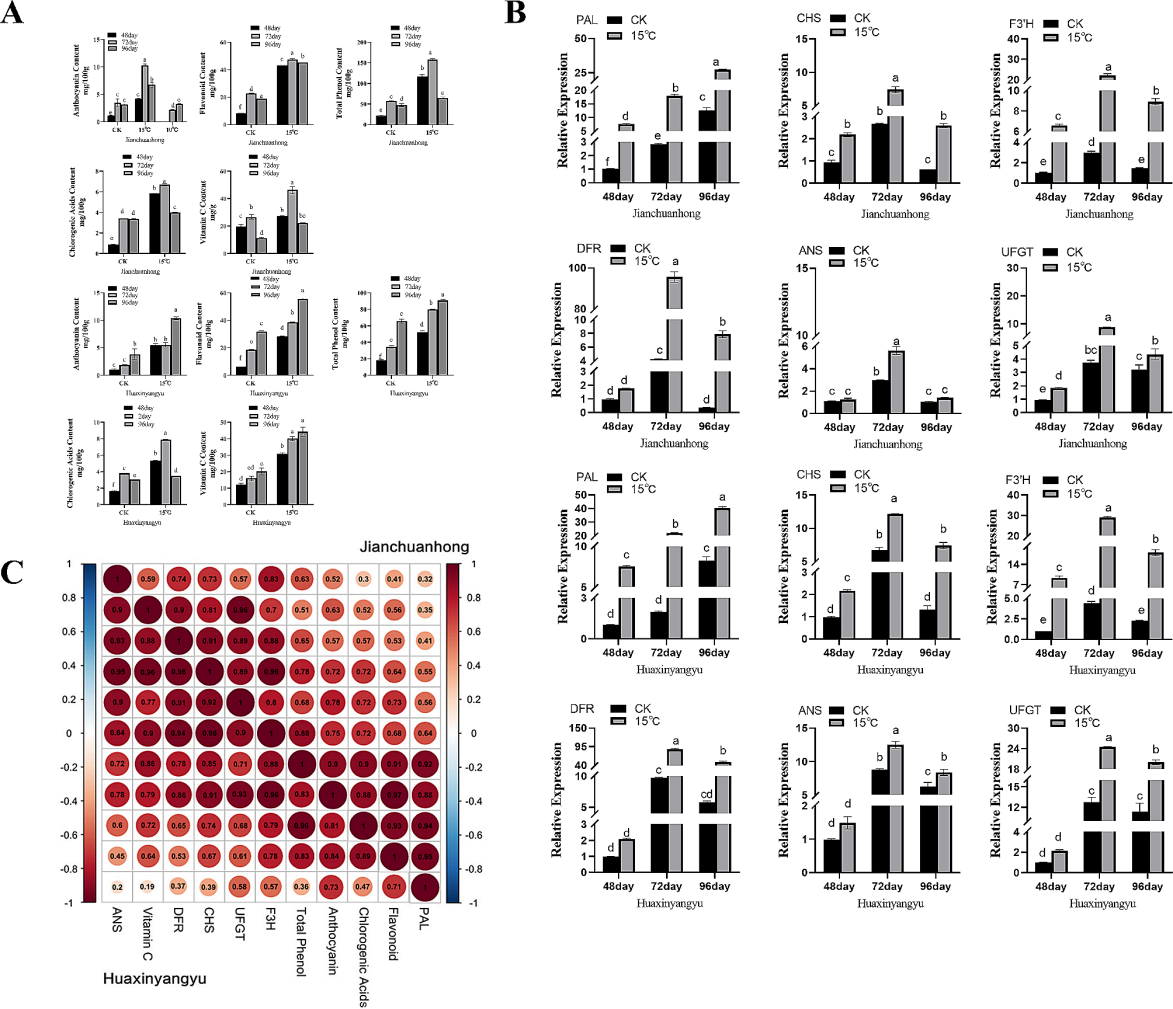
Analysis of antioxidant substances and anthocyanin synthesis structure gene expression levels in potato tubers and their correlation. **(a)** Effects of proper low-temperature treatment on the accumulation of antioxidant substances in Jianchuanhong tubers and Huaxinyangyu tubers. **(b)** Relative expression levels of structural genes involved in the anthocyanin synthesis pathway in two potato tubers. **(c)** Correlation analysis of physiological indexes and structural genes of potato tubers. Red represents a positive correlation, and blue represents a negative correlation. The data are the mean ± SD; *n* = 3; the larger the value, the more significant it is (*P* < 0.05)

We determined the content of antioxidant substances in the tubers of the two potato varieties (Fig. 3A). Compared with those at other temperatures, the tubers of these two varieties appeared darker at 15 °C (Fig. 1B), indicating that appropriate low-temperature treatment significantly promoted the accumulation of secondary metabolites in their tubers. Our results demonstrated that the production rates of total phenols, flavonoids, anthocyanins, chlorogenic acid, and vitamin C in Jianchuanhong tubers exhibited a noticeable trend of initially increasing and then decreasing over time, peaking at 72 days. On the other hand, in the Huaxinyangyu tubers, there was a continuous increase in the change in and accumulation antioxidant substances, reaching the peak at 96 days, with the fastest accumulation rate observed from 72 days to 96 days. Only chlorogenic acid showed a similar trend to that of Jianchuanhong, reaching its peak at 72 days.

Potato tubers are rich in vitamin C and are one of the best raw materials for extracting vitamin C [65]. There is a highly significant positive correlation between the dry matter content of potato tubers and the vitamin C content [66], which is one of the measures for judging the quality of potato tubers. Appropriate low temperatures can promote the accumulation of some nutrients. Elevated levels of ASA metabolism lead to increased tolerance to cold stress in plants [20]. Studies have confirmed that the ASA metabolic system in potato leaves is stimulated in the early stages of low-temperature treatment, and the ASA content first increases, peaks, and then decreases [38]. Vitamin C content during potato tuber growth because it is present in both reduced (ascorbic acid) and oxidized (dehydroascorbic acid) forms, dehydroascorbic acid is gradually reduced to ascorbic acid as the tubers mature [67] because the vitamin C content change appears as an increase with growth and development before the tubers mature. Later, during tuber development, due to the senescence of the aboveground plants, the physiological metabolic consumption of the tubers intensifies, and thus the vitamin C content decreases with increasing maturity [68]. Vitamin C is a water-soluble vitamin and an antioxidant [69]. Potato tubers are rich in vitamin C and are one of the best raw materials for extracting vitamin C [70]. There is a highly significant positive correlation between the dry matter content of potato tubers and the vitamin C content [66], which is one of the measures for judging the quality of potato tubers. Appropriate low temperatures can promote the accumulation of some nutrients. Elevated levels of ASA metabolism lead to increased tolerance to cold stress in plants [20]. Studies have confirmed that the ASA metabolic system in potato leaves is stimulated in the early stages of low-temperature treatment, and the ASA content first increases, peaks, and then decreases [38].

Chlorogenic acid (CGA) is a natural phenolic antioxidant. Under a low-temperature environment, plants exhibit an increase in the content of chlorogenic acid, which helps plants cope with adverse growth environmental stresses [71]. Previous researches have shown that the content of chlorogenic acid can increase in tubers by activating the phenylpropanoid pathway and promoting the relative expression of *PAL*, a structural gene [72]. The content of chlorogenic acid in some solanaceous crop species, such as tomato and sweet potato, tends to increase after low-temperature treatment [73, 74]. Pigmented potato has higher phenolic acid content than ordinary varieties, and chlorogenic acid accounts for the highest proportion of phenolic acids, thus possessing higher biological activity and stronger antioxidant performance [75].

Phenolic compounds constitute an important class of plant secondary metabolites that play a key role in many physiological reactions that occur during the plant life cycle. Plants exhibit increased contents of polyphenols, such as flavonoid compounds and chlorogenic acid, under abiotic stress conditions, which helps plants cope with adverse growth environmental stresses [70, 71]. The anthocyanin biosynthetic pathway has been relatively thoroughly studied in plants, all of which use phenylalanine as a direct target. Structural genes directly encode enzymes in the anthocyanin biosynthesis pathway and thus affect anthocyanin metabolism [76]. Moreover, low temperature has also been shown to induce an increase in the chlorogenic acid content [71].

Our results indicate a significant increase in the contents of total phenols, flavonoids, anthocyanins, and chlorogenic acid in the tubers of both potato varieties with prolonged appropriate low-temperature treatment during potato tuber formation. Previous studies have highlighted that the phenylpropanoid biosynthesis pathway becomes activated under abiotic stress conditions such as low temperature. This activation enables plants to synthesize higher quantities of phenolic compounds such as flavonoids and chlorogenic acid by activating key biosynthetic enzymes and upregulating key genes [67], ultimately resulting in the protection of plant cells. Therefore, due to the potato plant’s own defense response to low-temperature conditions, tuber tissue can produce more antioxidant substances by regulating the activities of related structural genes and key enzymes.

### Fluorescence quantitative PCR analysis based on low-temperature treatment

To further elucidate how low temperature influences anthocyanin accumulation, we conducted an analysis of the expression levels of structural genes involved in the anthocyanin biosynthesis pathway. The results from q-RT-PCR revealed that various downstream structural genes were upregulated to varying degrees in potato tubers subjected to appropriately low temperatures compared to those in the control group (Fig. 3B). In the two pigmented potato varieties, the relative expression of *StANS*, *StUFGT*, *StDFR*, *StF3’H*, *StPAL*, and *StCHS* peaked after 72 days of treatment. Notably, among Jianchuanhong and Huaxinyangyu, *StPAL*, *StF3’H*, and *StDFR* exhibited the most significant changes, with *StPAL* increasing by 6.4 and 11 times, StF3’H increasing by 7.3 and 6.6 times, and *StDFR* increasing by 22.53 and 9 times, respectively. The physiological data also revealed that the anthocyanin content peaked in Jianchuanhong after 72 days, while in Huaxinyangyu, it peaked at 96 days, with maximum accumulation observed from 48 to 72 days. These results align with the anthocyanin physiological data of the two pigmented potato varieties, suggesting that low-temperature treatment can regulate anthocyanin accumulation through the upregulation of *StANS*, *StUFGT*, *StDFR*, *StF3’H*, *StPAL*, and *StCHS* expression. However, the specific mechanism requires further exploration.

Recent studies have shown that the biosynthesis of anthocyanins is completed by the coordinated expression and regulation of a series of structural genes and regulatory genes [77, 78]. In essence, phenylalanine is used as a direct precursor and is catalyzed by a series of reactions involving enzymes encoded by structural genes [79]. Low temperature can upregulate the expression of structural genes (*StPAL*, *StDFR*, *StANS* and *StUFGT*) involved in anthocyanin synthesis [80]. Xu et al found that low temperature could upregulate *SlDFR* and *Sl F3‘5’* in tomato to induce anthocyanin synthesis [81]; Leon’s study showed that *F3‘5’ H* and *DFR* significantly increased in sweet pepper stems under low-temperature treatment [82]; Zhou et al.‘s Arabidopsis overexpressing the eggplant smcbf gene showed a trend of increasing anthocyanins with structural genes under low temperature [83].

### Correlation analysis of physiological indexes and structural genes of potato tubers

We conducted a correlation analysis on the physiological indicators of the tubers of both pigmented potato varieties and the structural genes related to anthocyanin synthesis (Fig. 3C). The results revealed significant positive correlations between the anthocyanin content in Jianchuanhong and the total phenol content, while the anthocyanin content in Huaxinyangyu showed a significant positive correlation with the flavonoid content. In terms of structural genes, *StF3’H*, *StUFGT*, *StCHS* and *StDFR* in Jianchuanhong were significantly positively correlated with anthocyanin content, while only *StPAL* and *StF3’H* in Huaxinyangyu were significantly positively correlated with anthocyanin content. Additionally, the anthocyanin content and vitamin C content in both varieties exhibited significant positive correlations, and their change trends were also significantly positively correlated, while no significant correlation was observed with the change trend of chlorogenic acid.

We also observed suberization in the tubers of both potato varieties after low-temperature treatment, with Huaxinyangyu showing more pronounced suberization (Fig. 1B). Furthermore, the relative expression of *StPAL* was higher in Huaxinyangyu (Fig. 3C). Previous studies have demonstrated that phenylpropanoid metabolism is induced during potato suberization, leading to increased measurable *StPAL* activity following the onset of suberization in potato tubers [84, 85].

## Conclusion

The results showed that long-term low-temperature treatment also significantly changed the activities of various enzymes in potato leaves, increasing plant resistance to long-term low-temperature environment. During the formation of potato tubers, with the extension of appropriate low-temperature treatment time, the content of secondary metabolites in the tubers of the two potato varieties increased significantly, activating the phenylpropanoid biosynthesis pathway under abiotic stress conditions such as low temperature, which enables plants to synthesize more phenolic compounds by activating key biosynthetic enzymes and upregulating key genes and ultimately protecting plant cells. Therefore, due to the defense response of potato plants to low-temperature conditions, potato tubers can produce more secondary metabolites by regulating the activities of structural genes and key enzymes. The results of this study are of great significance for the preliminary exploration of the effect of temperature on the added economic value of pigmented potato tubers and provide a theoretical basis for the selection of the growth environment for pigmented tuber-producing potato varieties.

## Data Availability

No new sequencing data were generated in the current study.

## Data availability

The data that support the findings of this study are available from the fist author, Bi-Cong Chen, upon reasonable request.
